# Human plasma kallikrein: roles in coagulation, fibrinolysis, inflammation pathways, and beyond

**DOI:** 10.3389/fphys.2023.1188816

**Published:** 2023-08-30

**Authors:** Guacyara Motta, Luiz Juliano, Jair Ribeiro Chagas

**Affiliations:** ^1^ Departamento de Bioquímica, Escola Paulista de Medicina, Universidade Federal de São Paulo, São Paulo, Brazil; ^2^ Departamento de Biofisica, Escola Paulista de Medicina, Universidade Federal de São Paulo, São Paulo, Brazil

**Keywords:** bradykinin, kininogen, kallikrein, proteolysis, cell biology, inflammation, inhibitors

## Abstract

Human plasma kallikrein (PKa) is obtained by activating its precursor, prekallikrein (PK), historically named the Fletcher factor. Human PKa and tissue kallikreins are serine proteases from the same family, having high- and low-molecular weight kininogens (HKs and LKs) as substrates, releasing bradykinin (Bk) and Lys-bradykinin (Lys-Bk), respectively. This review presents a brief history of human PKa with details and recent observations of its evolution among the vertebrate coagulation proteins, including the relations with Factor XI. We explored the role of Factor XII in activating the plasma kallikrein–kinin system (KKS), the mechanism of activity and control in the KKS, and the function of HK on contact activation proteins on cell membranes. The role of human PKa in cell biology regarding the contact system and KSS, particularly the endothelial cells, and neutrophils, in inflammatory processes and infectious diseases, was also approached. We examined the natural plasma protein inhibitors, including a detailed survey of human PKa inhibitors’ development and their potential market.

## 1 Brief history

Human plasma kallikrein [E.C.3.4.21.34] (PKa), initially described by Hathaway et al. (1965), was later further characterized (Hathaway and Alsever, 1970) and then named the Fletcher factor (after the surname patient-family name). The detailed characterization and isolation of PKa were the first approaches (Moriya et al., 1965; Colman et al., 1969; McConnell and Mason, 1970; Sampaio et al., 1974).

Wuepper and Cochrane (1972) reported the effect of PKa on coagulation *in vitro*, and the studies with enzymes purified from human plasma established that PKa acts as a proteolytic activator of factor XII (FXII) (Revak et al., 1977).

Plasma deficiency in PKa presented alterations in coagulation tests, kinin release, fibrinolytic activity after plasma activation by kaolin, and chemotactic activity, all of them characterized by correction with plasma prekallikrein (PK), the zymogen form of PKa, that functions as a kinin releaser (Wuepper, 1973), chemotactic factor, and Hageman factor (FXII) activator (Weiss et al., 1974; Nakayasu and Nagasawa, 1979).

**FIGURE 1:**
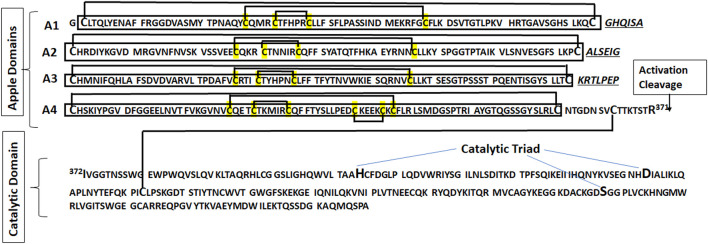
The sequence of PKa shows the apple domains A1 through A4 that constitute the heavy chain, linked to the catalytic domain (light chain) by a disulfide bound. The apple domains A1, A2, and A3 contain six cysteine residues linked by three disulfide bridges (C1-C6, C2-C5, and C3-C4; the numbers indicate the order of each cysteine in sequence). The apple domain A4 contains a fourth disulfide bridge. The connecting sequences of the apple domains are in italics.

Detailed studies demonstrated the hepatic synthesis of PK and its secretion into the blood as a single polypeptide chain glycoprotein with two molecular species of molecular masses (MW) 88 kDa and 86 kDa (Nagase and Barrett, 1981), respectively, due to the differences in glycosylation extension. PK is activated to PKa by Factor XIIa (FXIIa), and the two species consist of a heavy chain (amino-terminal), with an MW of 43 kDa, and a light chain (carboxy-terminal), with two variants of MW 36 kDa or 33 kDa and occurring in equimolar amounts. The light-chain region contains the enzymatic active site, and hydrolyzes oligopeptide substrates, protein substrates, and the zymogen form of FXII. Nevertheless, the heavy chain participates in binding to high-molecular weight kininogen (HK), providing its optimal cleavage, and in the surface-dependent activation of coagulation (Mandle et al., 1976; Fisher et al., 1982; van der Graaf et al., 1982). Figure 1 shows the sequence and structure of PKa after activation by hydrolysis at the R371–I372 peptide bond, and a schematic structure from a high-resolution 1.3-Å structure of full-length PKa in the active conformation, including the apple domains, has been reported in detail (Li et al., 2019).

Relevant observations about the physiological control of PKa are its clearance from circulation by hepatocytes (Borges et al., 1981; Borges and Kouyoumdjian, 1992; Kouyoumdjian et al., 2005), inhibition by the plasma-circulating protein inhibitors discussed in section 7.1, and inactivation by proteolysis (Motta et al., 1992). In addition to the contact system of the intrinsic coagulation pathway, PKa plays a role in pathophysiological processes as a potent pro-urokinase (pro-uPA) activator (Ichinose et al., 1986), in the renin–angiotensin system as a prorenin activator (Hsueh et al., 1981; Almeida et al., 2000), as an activator of C3 convertase (DiScipio, 1982; Irmscher et al., 2018), and as factor B of the complement system (Hiemstra et al., 1985).

## 2 Genomic structure and evolution

The genomic structure of PK (also KLKB1) (Beaubien et al., 1991; Yu et al., 2000) comprises a single gene in the human genome that maps to chromosome 4 (q34-q35), and hepatocytes are the local center of the PK gene transcription. However, it also occurs at different levels in non-hepatic tissues, but circulating PK in plasma is obtained essentially from the liver. It would be reasonable to assume that PK synthesized in extrahepatic tissues presents special functions in or close to their synthesis locations (Ciechanowicz et al., 1993; Hermann et al., 1999; Neth et al., 2001; Neth et al., 2005).

The evolution of the plasma kallikrein–kinin system (KKS) and Factor XI (FXI) described by Ponczek et al. (2008) and later in more detail by Ponczek et al. (2020) showed the KKS appeared in lobe-finned fish, the ancestors of all land vertebrates. Duplication of the gene for PK occurred during mammalian evolution, resulting in FXI changing independently of the KKS in placental mammals.

A significant observation related to the evolution of the KKS was the molecular studies of kalliklectin from channel catfish (*Ictalurus punctatus*), a fish-specific lectin containing eight apple domains, with structures similar to those of mammalian PK/FXI but without proteolytic activity (Tsutsui et al., 2021). Recently, the same group reported genomic sequences encoding a protein with apple and serine protease domains in a few cartilaginous and bony fish species by bioinformatic analysis and also purified, by mannose-affinity chromatography. Two ∼70 kDa proteins from the blood plasma of Ictalurus punctatus containing internal amino acid sequences were mapped onto possible PK/FXI-like sequences, which contains the typical cleavage site of mammalian PK and FXI, indicating protease activity (Tsutsui et al., 2023). Another new finding in this report is that catfish PK/FXI-like proteins have lectin activity.

Table 1 shows the PK, FXI, FXII, and HK appearances in the representative classes in the evolution, as reported by Ponczek et al. (2020), in which we added the recent contribution of Tsutsui et al. (2023) that can provide alternative evolutionary scenarios.

**TABLE 1:** Evolution of vertebrate coagulation proteins. The symbols in the columns indicate if a gene for the respective proteins was identified (+) or not identified (−) in genomic analyses. Data from Ponczek et al. (2020); Tsutsui et al. (2023). Table 1 was reprinted from BlooEsterl W, Bhoola KDd Adv. 4(24) Dec 22. Ponczek MB, Shamanaev A, LaPlace A, Dickeson SK, Srivastava P, Sun MF, Gruber A, Kastrup C, Emsley J, Gailani D. The evolution of factor XI and the kallikrein–kinin system. Pages 6135-6147, 2020. Doi:10.1182/bloodadvances.2020002456, with permission from Elsevier, License 5600360083462, Copyright Clearance Center’s RightsLink^®^. RightsLink^®^.

Class	Example organism	PK	FXI	FXII	HK
Jawless fish (Agnatha)	Sea lamprey	**-**	**-**	**--**	**-**
Cartilaginous fish (Chondrichthyes)	Whale shark	**-**	**-**	**-**	**-**
Actinopterygii	Zebrafish (ray-finned fish)	**-**	**-**	**-**	**-**
	Channel catfish (*Ictalurus punctatus*)	**+**	**+**	**-**	**-**
Lobe-finned fish (Sarcoptyrigii)	Coelacanth	**+**	**-**	**-**	**+**
	West African lung fish	**+**	**-**	**+**	**+**
Amphibians	African clawed frog	**+**	**-**	**+**	**+**
Reptiles	American alligator	**+**	**-**	**+**	**+**
Birds	Chicken	**+**	**-**	**-**	**+**
Egg-laying mammals (Monotremes)	Duck-billed platypus	**+**	**+**	**+**	**+**
Pouched mammals (Marsupials)	Opossum	**+**	**+**	**+**	**+**
Placental mammals (Eutherians)	Human	**+**	**+**	**+**	**+**
Placental diving mammals (Cetaceans)	False killer whale	**-**	**+**	**-**	**+**

Relevant evolution occurred in the A4 domain of tetrapod PKs, including humans, with the additional Cys321–Cys326 disulfide bond (Figure 1) (McMullen et al., 1991), which is also present in the lungfish. The cysteine C^326^ in PK-A4 resulted in an extra disulfide bridge in PK-A4, and the substitution by G^326^ in FXI-A4 resulted in a free -SH group of C^321^ responsible for FXI dimerization (Cheng et al., 2003). Another relevant acquisition of FXI is proline at position 368 (P^368^), close to the cleavage site (R^369^-I^370^), for the activation to FXIa, which turned out to be susceptible to thrombin that requires proline at the P_2_ position (Schechter and Berger, 1967; Gallwitz et al., 2012), that amplifies the coagulation process and integrates the extrinsic and intrinsic pathways, as reviewed in Mohammed et al. (2018) and recently revisited in Barroeta et al. (2023).

## 3 Cell biology of the plasma kallikrein–kinin system

Two extensive and comprehensive reviews of biochemistry and interactions of the blood contact activation system (Colman and Schmaier, 1986) and bioregulation of kinins (Bhoola et al., 1992) stressed the structural and functional characterization of PKa and its chains, along with its role related to coagulation, fibrinolysis, complement, and inflammation. PKa potentiates platelet aggregation induced by ADP, collagen, and adrenaline (Cassaro et al., 1987; Ottaiano et al., 2017).

**FIGURE 2:**
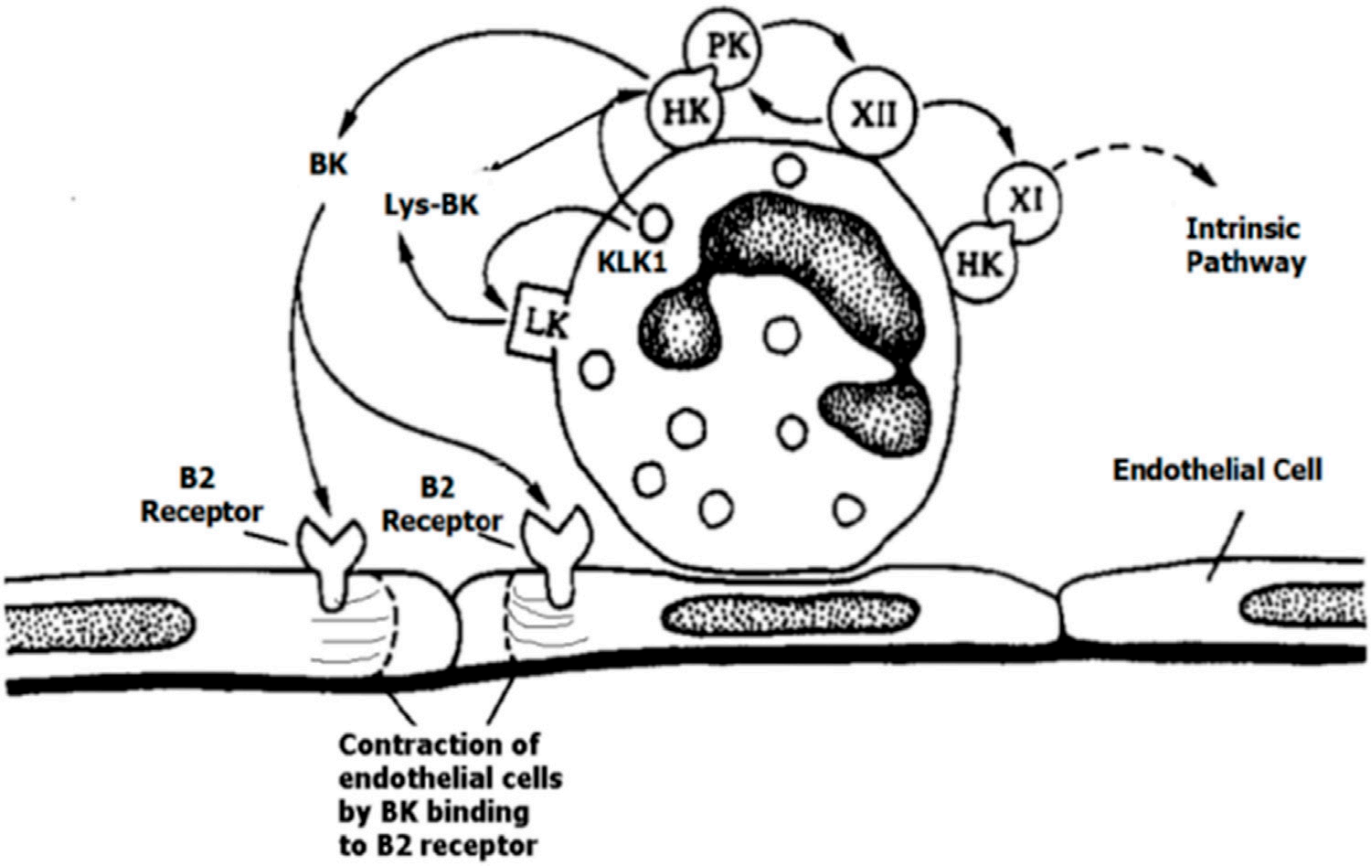
Setting of the contact-phase factors HK, PK, FXI, and FXII on neutrophils, with the reciprocal activation of PK—HK with bradykinin (BK) and Lys-BK released by tissue kallikrein 1(KLK1), adapted with permission from Henderson et al.,1994. Figure 2 was reprinted from Blood 84(2) Jul 15. Henderson LM, Figueroa CD, Muller–Esterl W, Bhoola KD. Assembly of contact-phase factors on the surface of the human neutrophil membrane, pages 474-82, 1994. Doi: 10.1182/blood.V84.2.474.474, with permission from Elsevier, License #5597371352787, Copyright Clearance Center’s RightsLink^®^.

The study of the KKS proteins in cell biology was a landmark between contact and KKSs despite their participation in both processes (Colman and Schmaier, 1997). The outer face of the neutrophil membrane binds the contact-phase proteins (HK, PK, FXI, and FXII) (Figure 2), as demonstrated by immunolocalization techniques (Henderson et al., 1994).

The endothelial cells and platelet membranes assemble HKs; however, on endothelial cells and the extracellular matrix, PK binds mainly through assembled HK, and this interaction allows PKa formation independent of the FXII assembly on the cell membrane (Motta et al., 1998; Schmaier, 1998; Motta et al., 2001; Motta and Tersariol, 2017). Heat-shock protein 90 (Hsp-90) (Joseph et al., 2002) and prolylcarboxypeptidase (PRCP) (Shariat-Madar et al., 2002) are two other activators of PK besides FXIIa.

PKa plays relevant roles in inflammation, as reviewed with appropriate citations (Schmaier, 2016). Thrombosis and inflammation are present in many tissue injuries with multiple crossed-ways and interfaces between them (Wu, 2015), and with extensive participation of neutrophils (Mortaz et al., 2018), including all types (Rosales, 2018).

## 4 Functions of the structural domains of kininogens

Muller–Ester’s and Schmaier’s groups identified the portions of HK that bind to the cell surface using monoclonal antibodies against different parts of HK (Kaufmann et al., 1993; Hasan et al., 1994) and the discontinuous binding site for HK on the PK heavy chain (Hock et al., 1990).

The HK interaction with the cell surface has been well characterized (Schmaier and McCrae, 2007). The HK presents six domains from its N-terminal portion (D1-D6); each domain plays a particular function that characterizes the whole molecule as a multifunctional protein. The domains D5(His-Gly-Lys rich), D4(BK), and D3 (cysteine protease inhibitor) allow the molecule to interact with the cell membrane. Nevertheless, after HK cleavage and bradykinin (BK) release, the intact molecule turns to BK-free-HK (HKa) (Lalmanach et al., 2010).

The organization of HK in six domains, as described previously, with clearly defined functions, has been well-established and reviewed in many publications. Nonetheless, determining its tridimensional (3D) structure is still challenging. The size of the protein, its multi-structural organization, and heavy glycosylation seem to be barriers to obtaining this goal. The structural changes that probably follow BK release or the acquired conformation upon an interaction with PK or ligands in the endothelial surface should be equally challenging to obtain. As recently explained by Ponczek, 2021), neither crystallography nor NMR methods provided published structures of HK. As suggested, a probably helpful approach, given the recent advances in the technique, would be the utilization of cryo-electron microscopy (cryoEM) not only for the determination of the 3D structure of HK itself but also for its many known physiologically relevant complexes with protein or non-protein molecules. For example, the interaction of HK with glycosaminoglycans and Zn^2+^ (Gozzo et al., 2011), which changes the kinetics of BK release by the PK from HK, would be attractive structural targets for cryoEM.

PK is an unusual plasma protease as it does not recruit its principal substrate, HK, upon activation. However, it instead circulates as a zymogen in a tightly bound but inactive PK–HK complex (Mandle et al., 1976), whose structure consists of HK binding to discontinuous sites of PK at its N-terminal apple domain in the rank order of binding affinity for kininogen of A2 > A4 = A1 > A3, A2 domain being essential for binding (Renné et al., 1999). It is important to mention that regions of the A3 domain have increased surface exposure in PKa compared to PK, indicating conformational changes upon activation (Li et al., 2019).

PK and FXI, through their heavy chain, circulate bound to domain 6 of HK (Vogel et al., 1990; Kunapuli et al., 1993). Either FXI or PK interacts via the apple domain A2 on the HK sequence (F^582^NPISDFPDT^591^); nevertheless, in this interaction with HK, PK has the second exosite in the apple domain A1, but FXI interacts with a unique pocket formed between A2 and A3 domains (Li et al., 2023).

The membrane-binding proteins of both HK and HKa (activated kininogen) on endothelial cells include the globular domains of the complement factor C1q receptor (gC1qR), urokinase plasminogen activator receptor (uPAR), and cytokeratin 1 (CK1), and the affinity levels among them are gC1qR > CK1 > soluble uPAR, indicating that gC1qR is dominant for binding. uPAR is an essential membrane-binding protein for differential binding and signaling between HK and HKa (Pixley et al., 2011).

Proteoglycans (PGs) are molecules expressed by all cells and found in all extracellular matrices (ECMs), consisting of a protein core onto which one or more of the negatively charged polysaccharide compounds, glycosaminoglycan (GAG) chains, are covalently attached (Karamanos et al., 2018). Heparan sulfate works as a putative receptor of either HK or PK, alone or in complex with each other, mediating the endocytosis and activation of HK and PK (Motta and Tersariol, 2017).

## 5 Kallikrein–kinin system as a multifunctional system

The two forms of plasma kininogens, HK and LK (low-molecular weight kininogen), are the products of a single gene that maps to 3q26-qter. The single kininogen gene of 11 exons of 27 kb produces a unique mRNA for HK and LK by alternative splicing. HK and LK share the coding region of the first nine exons, a part of exon 10 containing the BK sequence, and the first 12 amino acids after the carboxy-terminal sequence of BK. Exon 11 codes for a unique 4-kD light chain of LK, and the complete exon 10 contains the entire coding sequence for the unique 56-kD light chain of HK. So, the splice variant LK is 409 amino acids in length, and the principal difference is the loss of HK domain 6 (Colman and Schmaier, 1997).

The tissue and PKa, KLK1 and PKa, belong to the same family of serine proteases and show high sequence similarity in the region encoding the trypsin-like domain. Interestingly, the comparative genomics and phylogenetic analysis argue for a previous origin of PKa than KLK1. Kallikreins exert their major physiological roles by acting as proteases. Still, the expression of KLK protein isoforms, without the protease activity, may indicate that KLKs have other functions, at least in humans (Koumandou and Scorilas, 2013).

### 5.1 Role of bradykinin

The kinins, initially BK and, by the action of carboxypeptidases, desArg^9^BK, are the end active peptides generated after the activation of PK to PKa. Human KLK1, unlike PKa, cleaves LK to generate Lys-BK (also kallidin), which is converted to BK by a second aminopeptidase cleavage (Pathak et al., 2013). The pathophysiological and beneficial physiological effects of kinins in various cardiovascular disorders such as hypertension, ischemic heart disease, left ventricular hypertrophy, ventricular remodeling, congestive heart failure, and diabetic conditions occur by the stimulation of BK receptors (B2 and B1) (Heitsch, 2003; Sharma, 2003; Marceau et al., 2020).

Recently, comprehensive reviews have extensively dealt with many aspects of BK and desArg^9^BK generation; its metabolism by endo, carboxy, and amino peptidases; and the multitude of actions attributed to both peptides in many cell models, tissues, and organs (Lalmanach et al., 2010; Campbell, 2013; Motta et al., 2018). The role of BK in angioedema as the B2BK receptor agonist has been unequivocally established (Hofman et al., 2016; Schmaeir, 2018; Rex et al., 2022; Gailani, 2023) through the increase in vascular permeability, but also desArg^9^BK, the B1BK receptor agonist, seems to be relevant as these receptors modulate neutrophil trafficking (Araújo et al., 2001). Neutrophil elastase release, among other effects (Stuardo et al., 2004), is one of the factors able to inactivate the C1-esterase inhibitor (C1-INH) or cleave FXII, accelerating FXII activation by PKa (de Maat et al., 2019; Ferrara et al., 2021).

A recent review highlights multiple mechanisms for the increased generation of kinins or their slowed inactivation, resulting in angioedema (Gailani, 2023). Of significant interest is the recently described mutation on the Glu^311^-plasminogen that turns plasmin, which is able to release BK from HK and LK at considerably higher rates than wild-type plasmin. Considering that LK concentrations are two to four times higher in plasma than HK, all this evidence indicates that LK, previously a not-so-relevant player in BK generation in angioedema, can be an important actor.

### 5.2 Role of FXII in activation of KKS

FXII, the zymogen of the protease FXIIa, plays a role in BK-dependent angioedema and thrombosis by activating prekallikrein and factor XI zymogen (FXI). These activation processes are highly magnified when FXII binds to a surface through EGF1, gaining an open conformation that facilitates FXII activation. These processes are recently described and discussed in detail (Shamanaev et al., 2022; Shamanaev et al., 2023).

More recently, the crystal structure for analyzing factor XII, HK, or both binding to gC1qR has been solved. FXII-HK-gC1qR forms a ternary complex assembled in the presence of Zn^2+^, stimulating reciprocal FXII-PK activation by gC1qR (Kaira et al., 2020). The authors also compared the asymmetric binding onto gC1qR to multiple client proteins that can be colocalized by asymmetric binding onto the Hsp-90 dimer (Flynn et al., 2015). The extracellular Hsp-90 has been described as a chaperokine involved in inflammatory processes (Taha et al., 2019) and as a PK activator on the cell surface, as mentioned previously (Joseph et al., 2002).

## 6 Mechanisms of the KKS in diseases

It is relevant to emphasize that the initial contributions of the plasma KKS related to coagulation, fibrinolysis, and inflammation processes remain under investigation over the years (Kolte and Shariat-Madar, 2016; Schmaier, 2016; Simão and Feener, 2017; Oehmcke-Hecht et al., 2022). More recently, it has been shown that PKa could be a significant physiological activator of FIX (Kearney et al., 2021).

Genetic manipulation of the KKS in mice has allowed recognition of the physiological role of the KKS in health and disease (Girolami et al., 2014). Mice deficient in the PK gene (Klkb1^−/−^) show an alternative mechanism for thrombosis protection, in addition to reduced contact activation, mediated by increased receptor Mas, prostacyclin, Sirt1, and KLF4 (Stavrou et al., 2015).

### 6.1 Role in non-infectious diseases

In the central nervous system inflammatory processes, PKa acts as a neuromodulator by engaging PAR-2 and BK-B2 receptors (Jaffa et al., 2021), and the complex biology of kinins also affects cancer progression (Deepak et al., 2022). In liver injury, PKa cleaves the transforming growth factor (TGF)-beta 1 (Li et al., 2018; Ahmed et al., 2021).

#### 6.1.1 Diabetes mellitus (PKa activation of PAR1/2)

In addition to neutrophils, endothelial cells, and platelets, the contact system proteins bind to vascular smooth muscle cells (VSMCs) (Fernando et al., 2005). In the aortic VSMC, PKa activates the ERK1/2 mitogen-activated protein kinase cascade with the stimulation of ADAM 17 (a disintegrin-metalloprotease) via a PAR1/2 receptor-dependent mechanism, without the involvement of BK receptors. The BK-independent PKa action may regulate vascular responses in pathophysiologic states, such as diabetes mellitus (Abdallah et al., 2010).

#### 6.1.2 Ocular and cerebral disorders (BK release by PKa)

The connection of fibrinolysis and the KKS at several levels supports understanding hereditary angioedema and other forms of vascular permeability mediated by BK (Tomita et al., 2012; Kaplan and Maas, 2017).

In the eye, the consequences of diabetes are microvascular abnormalities, the proliferation of retinal vessels, and increased retinal vascular permeability. The involvement of either kallikrein–kinin or renin–angiotensin systems has been reported in diabetic retinopathy, glaucoma, uveitis, diabetic macular edema, and age-related macular degeneration. In this way, the breakdown of the blood–retinal barrier function, which leads to increased retinal vascular permeability, results in significant alterations in the biochemical components of intraocular fluids and diffusion of blood-circulating factors into the interstitial retinal space and vitreous (Abdulaal et al., 2016; Igić, 2018; Othman et al., 2021).

In vitreous patients, carbonic anhydrase-I (CA-I), an intracellular enzyme, suggests retinal hemorrhage and erythrocyte lysis (Gao et al., 2007). CA-I induces alkalinization of vitreous, increasing the PKa activity and generating factor XIIa, revealing a new pathway for contact system activation. In addition, these authors showed the presence of extracellular CA-I inside either the blood–retinal or blood–brain barrier that can induce vasogenic edema. The blood–brain barrier breakdown seems to exist in patients with temporal lobe epilepsy (TLE) due to KKS activation (Simões et al., 2019).

### 6.2 Role in infectious diseases

The fibrin frames generated within the microvessels by contact systems capture microorganisms, reducing their spread through the whole organism, and facilitate activated leukocytes’ functions (Stark and Massberg, 2021). This process results from converging platelet-generated thrombogenesis with the activated neutrophils and monocytes. This intrinsic path results from the autoactivation of factor XII (FXIIa) by negatively charged substances, such as platelet-derived polyphosphates and DNA, from neutrophil extracellular traps that are the contact platforms. Once functional, FXIIa cleaves PK, generating PKa, releasing BK from HK, and initiating inflammation.

BK induces vasodilation and increases microvascular permeability by activating the endothelial BK-B2 receptors. A second receptor for BK is BK-B1, which presents high affinity for des-Arg^9^-BK produced by GPI-linked carboxypeptidase M by removing the C-terminal arginine from BK.

Scharfstein and his group extensively reviewed these processes, focusing mainly on *Trypanosome cruzi* as the infectious agent and including extensive studies on the role of parasite cysteine proteases (Scharfstein et al., 2012; Scharfstein et al., 2017; Scharfstein, 2018). A similar interplay between parasite cysteine proteases of *Leishmania donovani* and *Leishmania chagasi* also critically modulates inflammation and innate immunity in visceral leishmaniasis using the host kinin/B2 receptor activation pathway (Svensjö et al., 2006; 2014).

*Plasmodium chabaudi* and *Plasmodium falciparum* internalize and process plasma kininogen by falcipain-2 and falcipain-3, both cysteine proteases, releasing Lys-BK, BK, and des-Arg^9^-BK and resulting in hemodynamic alterations during acute malaria (Bagnaresi et al., 2012; Cotrin et al., 2013), as well as increasing the blood–brain barrier permeability (Silva et al., 2019).

The extensive inflammation process is a characteristic of the clinical evolution of periodontitis due to gingipain, the cysteine protease complex from the Gram-negative bacteria *Porphyromonas gingivalis,* which efficiently activates the KKS with direct kinin release and PK activation with subsequent BK release (Imamura et al., 1994; Monteiro et al., 2009).

Regarding virus infection, it is noteworthy that contact/KKS activation followed by BK-induced enhancement of DENV replication in the endothelium seems to underlie microvascular pathology in dengue (Coelho et al., 2021). The contact/intrinsic pathway contributes to the pathogenesis of the prothrombotic state in COVID-19 (Carvalho et al., 2021; Alfaro et al., 2022; Henderson et al., 2022). In completing this theme, we mentioned an opinion article about the clinical repercussions of Prof. Dr. Sérgio Ferreira on COVID-19 (Nicolau et al., 2020).

Zanelatto et al. (2022) recently showed that cathepsin B and PKa measured in the serum can be used to discriminate different stages of liver damage in HCV-infected patients as biomarkers to exclude hepatic fibrosis in the population.

## 7 Plasma kallikrein inhibitors

### 7.1 Natural plasma proteins—serpins

C1-esterase inhibitor (C1-INH), α2-macroglobulin, antithrombin-III (van der Graaf et al., 1983), protein C inhibitor (PCI) (España et al., 1989), and alpha-2 plasmin inhibitor (Saito et al., 1979) are reported as PKa inhibitors. All belong to the serine proteinase inhibitor family (serpins).

Serpins have been extensively studied, and excellent recent reviews deal with their action mechanism, improvements in specificity, and potential use as drugs for replacement therapies (Maas and de Maat, 2021; Bouton et al., 2023). Briefly, serpins are proteins acting as molecular traps to proteases, particularly but not exclusively serine proteases, whose “beacon” is their carboxy-terminal regions, usually called the reactive center loop (RCL) that keeps the serpin in a high-energy state. Once cleaved, the RCL, but before a water molecule promotes the deacylation of the enzyme, changes to a lower energy state, where the remaining RCL remains linked to the enzyme in a new, lower-energy state. This structure is then buried in the five-strand beta-sheet (ß sheet A) present in serpins. It forms a sixth strand that makes water access difficult, blocks deacylation, and distorts the enzyme’s active center, rendering it inactive.

C1-INH plays a fundamental role in PKa inhibition. As estimated from biochemical experiments, approximately 42% of PKa inhibition is due to C1-INH activity, and 50% could be linked to α2-macroglobulin (Schapira et al., 1982) in normal plasma. The remaining 8% of inactivation could be attributed to other protease inhibitors in the plasma. The complete framework is much more complex when considering other enzymes and inhibitors in the *in vivo* steady-state physiological or pathological conditions. Proteolytic enzymes from the immune cells, for example, neutrophil elastase, can be released from the polymorphonuclear and mast cells and alter C1-INH activity (Ferrara et al., 2021; Kajdácsi et al., 2021). Anionic glycosaminoglycans, like heparin, heparan sulfate, chondroitin sulfate, and even polyphosphates, can interact either with the inhibitors or with the enzymes and dramatically change the inhibition or interaction constants (Grover and Mackman, 2022), adding more complexity to these interactions, as classically demonstrated for the antithrombin, PKa, HK, and heparin interaction (Olson et al., 1993).

### 7.2 Development and market overview

PKa inhibitor (PKi) development followed a parallel trajectory, which is typical in discovering new drugs. Many clues indicated the role of PKa in hereditary angioedema (HAE) at the end of the fifties and beginning of the sixties, in the last century. The work by Landerman et al. (1962) is not only an elegant set of experiments and conclusions but also demonstrates the role of PKa and suggests that a “permeability factor (PF)" released from the plasma of patients diagnosed with HAE was probably kallidin. Logical treatment of these patients would be with replacement therapy of the inhibitor to the proteinase or by an inhibitor to the proposed polypeptide.

This comprehensive review (Schmaier, 2018) clearly shows that, in 1963, the puzzle of the etiology of HA was solved (Donaldson and Evans, 1963). Still, the emergence of the complement system and the role of C1-inhibitor (C1-INH) in it postponed the recognition of Landerman’s suggestions to the early seventies (Gigli et al., 1970).

PKa inhibitors were the first drugs to treat PKa disbalance present in HA and also in other conditions related to uncontrolled PKa activity, such as diabetic retinopathy, macular edema (Bhatwadekar et al., 2020), sepsis, surgical intercurrences observed in cardiopulmonary bypass (CPB) surgery and other conditions related to inflammatory diseases, thrombotic events, and vascular alterations (Bryant and Shariat-Madar, 2009).

Nonetheless, attention should be given to the proper production of BK, as explained by Regoli and Gobeil (2015) and recent reports relating the reduced PKa activity to diabetic nephropathy (Härma et al., 2020). As with many other proteinase inhibitors, at first, molecules found in nature, like Kunitz and Bowman–Birk inhibitors, were purified and characterized by chromatographic and structural tools that evolved during the ‘70s, the ‘80s, and later, from plants (Oliva and Sampaio, 2009). As far as 1975, aprotinin (Trasylol^®^), a serine proteinase inhibitor of animal origin, was assayed during extracorporeal circulation in an open-heart surgery (Nagaoka and Katori, 1975).

However, most of these inhibitors have low specificity, potentially induce immune reactions, and cannot be patented. Nonetheless, the scaffolds of those inhibitors were used in the future, in the 90s and beyond, to select more effective molecules. Other contributions made to understand the PKa specificities and potentially add to the design of new inhibitors were obtained from many groups. The development of sensible substrates, especially with quenched fluorescence properties, accelerated the usually slow determination of kinetic constants (Chagas et al., 1991) and could combine this technique with known interactions in natural inhibitors (Nunes et al., 2003).

The group of Yoshio Okada, at Kobe University, during the ‘90s and early 2003, searched for small peptidomimetic inhibitors based on the known specificities of PKa. PSI-527 was one of the PKa inhibitors that reached the clinical trial but was discontinued (Hashimoto et al., 2003). Meanwhile, high-throughput methods for ligand selection became available. Phage display of interaction regions with PKa from known ligands or scFv immunoglobulin regions, peptide libraries, and chemical libraries was used to search for more effective inhibitors with potential clinical use.

In 1996, a specific and selective PKa inhibitor was obtained, later named ecallantide, from a phage display selection/maturation system, an inhibitor based on a Kunitz-type scaffold, identified from the first Kunitz domain of human lipoprotein-associated coagulation inhibitor (LAC1-D1), also known as tissue factor pathway inhibitor-I or TFPI-I (https://www.creative-biolabs.com/DX-88-Library-Construction.html) (Ley et al., 1996).

Later, in 2003, a paper entitled “DX-88 and HAE: a developmental perspective,” where DX-88 was the ecallantide molecule, reported the positive results of clinical phase I studies. In 2008, a summary of the DX-88 development was published and indicated the size of the potential uses of C1-INH activity replacement with molecules other than C1-INH itself (Lehmann, 2008). At this point, it became clear that a race between pharma companies had started. In 2009, ecallantide was approved by the Food and Drug Administration (FDA). From 2008 to 2010, concentrates of C1-INH (Berinert licensed in Germany since 1985 and Cynrise) were FDA-approved. A recombinant C1-INH (Ruconest, Rhucin) was approved in Europe (2010) and the USA (2014).

Phage display libraries also generated monoclonal antibodies that specifically inhibit PKa, like lanadelumab (Kenniston et al., 2014), approved by the FDA in 2018.

As stated previously, C1-INH is a serpin. Historically, phage display libraries based on the serpin scaffold have successfully found specific inhibitors for proteases belonging to the KLK family (Cloutier et al., 2004; Felber et al., 2006). However, we do not find reports of libraries based on the serpin scaffold that could select specific PKa inhibitors. Likewise, no other phage display libraries were found based on Kunitz or Bowman–Birk inhibitors for PKa despite a structure published about at least one of these inhibitors being effective for PKa inhibition (Zhou et al., 2015). These libraries have not been tried or have not shown effective results yet.

At the same time, phage display and other methods have originated protein structures that are able to inhibit PKa. Synthetic chemists have performed much work to create successful new inhibitory molecules. Berotralstat (BCX7353) (Kotian et al., 2021), sebetralstat (Davie et al., 2022), ATN-249 (Attune Pharmaceuticals), KVD824 (KalVista Pharmaceuticals), THR-149 (Oxurion NV), RZ402 (Rezolute Bio), and VE-3539 and VE-4840 (Verseon Corp) (Xie et al., 2020) were included.

Cyclic or bicyclic peptide libraries expressed in phage display systems have been employed to select new high-affinity and specificity inhibitors of PKa, whose interactions are extended to sites far from the active site, opening the possibility to explore new intermolecular interactions with PKa (Teufel et al., 2018).

Many options are on the way, including orally active inhibitors and protocols aimed at acute, chronic, and prophylactic treatments linked to PKa inhibition disbalance. These new developments of synthetic molecules, their properties, and their uses have been the subject of recent comprehensive reviews (Busse and Christiansen, 2020; Xie et al., 2020; Caballero, 2021; Busse and Kaplan, 2022). Meanwhile, although none of the 3D PKa structures were available on the Protein Data Bank database until 2005, 14 structures have been deposited (some from the same complex at different resolutions), as summarized in Table 2, indicating the increased interest of pharma companies, a race in new developments on this class of inhibitors and a sure indication of an exciting market. Ecallantide in the USA had an estimated market of U$ 150 million in 2010 (Zuraw et al., 2010). A recent report of 2010 estimated the global plasma protease C1-inhibitor treatment market at US$ 3,289.5 million in 2020, projected to be US$ 10,603.4 million in 2027 (https://www.coherentmarketinsights.com/market-insight/plasma-protease-c1-inhibitor-treatment-market-4262).

**TABLE 2:** 3D structures of PKa available and deposited in the Protein Data Bank (PDB).

PDB ID resolution	Year	Title	Reference
8A3Q 1.801 Å	2022	Human Plasma Kallikrein in complex with 14W	McEwan, P.A. (2022) J Med Chem **65**: 13629–13644
7N7X 2.1 Å	2022	Crystal structure of BCX7353 (ORLADEYO) in complex with human plasma kallikrein serine protease domain at 2.1 Å resolution	Krishnan, R., Yarlagadda, B.S., Kotian, P., Polach, K.J., Zhang, W. (2021) J Med Chem **64**: 12453–12468
6O1G Download FileView File 2.2 Å	2019	Full length human plasma kallikrein with inhibitor BCX4161	Partridge, J.R, Choy, R.M. (2019) J Struct Biol **206**: 170–182
6O1S 1.7 Å	2019	Structure of human plasma kallikrein protease domain with inhibitor BCX4161 download FileView File	Partridge, J.R., Choy, R.M. (2019) J Struct Biol **206**: 170–182
5TJX 1.41 Å	2017	Structure of human plasma kallikrein	Partridge, J.R., Choy, R.M., Li, Z. (2017) ACS Med Chem Lett **8**: 185–190
5F8Z 1.5 Å	2016	The crystal structure of human Plasma Kallikrein in complex with its peptide inhibitor pkalin-1 CYS-PRO-ALA-ARG-PHE-M70-ALA-LEU-PHE-CYS (protein)	Xu, M., Jiang, L., Xu, P., Luo, Z., Andreasen, P., Huang, M. To be published
5F8T 1.55 Å	2016	The crystal structure of human plasma kallikrein in complex with its peptide inhibitor pkalin-2	Xu, M., Jiang, L., Xu, P., Luo, Z., Andreasen, P., Huang, M.
5F8X 1.55 Å	2016	The crystal structure of human plasma kallikrein in complex with its peptide inhibitor pkalin-3	Xu, M., Jiang, L., Xu, P., Luo, Z., Andreasen, P., Huang, M.
4ZOT 1.4 Å	2015	Crystal structure of BbKI, a disulfide-free plasma kallikrein inhibitor at resolution	Shabalin,I.G., Zhou, D., Wlodawer, A., Oliva, M.L.V. (2015) Acta Crystallogr F Struct Biol Commun **71**: 1055–1062
4OGY 2.1 Å	2014	Crystal structure of Fab DX-2930 in complex with human plasma kallikrein	Edwards,T.E., Clifton, M.C., Abendroth, J., Nixon, A., Ladner, R. (2014) J Biol Chem **289**: 23596–23608
4OGX 2.4 Å	2014	Crystal structure of Fab DX-2930 in complex with human plasma kallikrein	Edwards,T.E., Clifton, M.C., Abendroth, J., Nixon, A., Ladner, R. (2014) J Biol Chem 289: 23596–23608
2ANW 1.85 Å	2005	Expression, crystallization, and three-dimensional structure of the catalytic domain of human plasma kallikrein: Implications for structure-based design of protease inhibitors	Tang, J., Yu, C.L., Williams, S.R., Springman, E., Jeffery, D., Sprengeler, P.A., Estevez, A., Sampang, J., Shrader, W., Spencer, J.R., Young, W.B., McGrath, M.E., Katz, B.A. (2005) J Biol Chem 280: 41077–41089
2ANY 1.4 Å	2005	Expression, Crystallization, and the Three-dimensional Structure of the Catalytic Domain of Human Plasma Kallikrein: Implications for Structure-Based Design of Protease Inhibitors	Tang, J., Yu, C.L., Williams, S.R., Springman, E., Jeffery, D., Sprengeler, P.A., Estevez, A., Sampang, J., Shrader, W., Spencer, J.R., Young, W.B., McGrath, M.E., Katz, B.A.2005) J Biol Chem **280**: 41077–41089
7QOX	2019	Plasma kallikrein structure reveals apple domain disc-rotated conformation compared to factor XI	Li, C., Voos, K.M., Pathak, M., Hall, G., McCrae, K.R., Dreveny, I., Li, R., Emsley, J. (2019) J Thromb Haemost 17: 759–770

A patent application (WO2022197761A1) claims that the compounds of the invention may be therapeutically beneficial for treating or preventing various ophthalmic, cardiovascular, or cerebrovascular thromboembolic conditions in patients suffering from unstable angina, acute coronary syndrome, refractory angina, myocardial infarction, transient ischemic attacks, atrial fibrillation, strokes such as thrombotic stroke or embolic stroke, venous thrombosis, coronary and cerebral arterial thrombosis, cerebral and pulmonary embolism, atherosclerosis, deep vein thrombosis, disseminated intravascular coagulation, reocclusion or restenosis of recanalized vessels, hereditary angioedema, uveitis, posterior uveitis, wet age-related macular degeneration, diabetic macular edema, diabetic retinopathy, and retinal vein occlusion. These perspectives are well beyond the original purpose of C1_INH studies for treating HAE. Hopefully, in the face of such a potential range of diseases or pathologies and the consequent expected market, many innovations will be implemented in the field.
